# How to Decrease Suicide Rates in Both Genders? An Effectiveness Study of a Community-Based Intervention (EAAD)

**DOI:** 10.1371/journal.pone.0075081

**Published:** 2013-09-23

**Authors:** András Székely, Barna Konkolÿ Thege, Roland Mergl, Emma Birkás, Sándor Rózsa, György Purebl, Ulrich Hegerl

**Affiliations:** 1 Institute of Behavioural Sciences, Semmelweis University, Budapest, Hungary; 2 Department of Psychology, University of Calgary, Calgary, Canada; 3 Klinik und Poliklinik für Psychiatrie und Psychotherapie, Universitätsklinikum Leipzig AoR, Leipzig, Germany; 4 Department of Personality and Health Psychology, Eötvös Loránd University, Budapest, Hungary; McGill University, Canada

## Abstract

**Background:**

The suicide rate in Hungary is high in international comparison. The two-year community-based four-level intervention programme of the European Alliance Against Depression (EAAD) is designed to improve the care of depression and to prevent suicidal behaviour. Our aim was to evaluate the effectiveness of a regional community-based four-level suicide prevention programme on suicide rates.

**Method:**

The EAAD programme was implemented in Szolnok (population 76,311), a town in a region of Hungary with an exceptionally high suicide rate. Effectiveness was assessed by comparing changes in suicide rates in the intervention region after the intervention started with changes in national suicide rates and those in a control region (Szeged) in the corresponding period.

**Results:**

For the duration of the programme and the follow-up year, suicide rates in Szolnok were significantly lower than the average of the previous three years (p = .0076). The suicide rate thus went down from 30.1 per 100,000 in 2004 to 13.2 in 2005 (−56.1 %), 14.6 in 2006 (−51.4 %) and 12.0 in 2007 (−60.1 %). This decrease of annual suicide rates in Szolnok after the onset of the intervention was significantly stronger than that observed in the whole country (p = .017) and in the control region (p = .0015). Men had the same decrease in suicide rates as women. As secondary outcome, an increase of emergency calls to the hotline service (200%) and outpatient visits at the local psychiatry clinic (76%) was found.

**Conclusions:**

These results seem to provide further support for the effectiveness of the EAAD concept. Whilst the majority of suicide prevention programs mainly affect female suicidal behaviour, this programme seems to be beneficial for both sexes. The sustainability and the role of the mediating factors (social service and health care utilization, community attitudes about suicide) should be key points in future research.

## Introduction

Hungary had the highest suicide rate in the world in the early nineteen eighties (44.9 per 100,000 in 1980). Although the rate has decreased since 1998, it is still among the ten highest in the world. The high prevalence thus goes back several decades, and the numbers remained largely constant through Hungary's major economic and political changes [1]. The overall national suicide rate in 2010 was 24.92 per 100,000 of population, but differed considerably by gender: 37.48 for men and 8.47 for women [2]. The transition in Hungary from socialism to market economy and democracy may have left suicide figures substantially unchanged, but it has had an effect on morbidity and mortality, rates, and much more strongly among males than females; depression was found to be a major risk factor of premature morbidity and mortality in the male population [3]–[6]. A four-year longitudinal observational study carried out between 2002 and 2006 found that 24% of men in the 40–69 year-old population who died during the study period were severely depressed at baseline, and only 6% of them were treated for depression [7].

The introduction of evidence-based depression and suicide prevention programmes is especially important in Hungary. Since suicide rates here are more than four times higher among men than among women, it is extremely important to find interventions capable of decreasing male as well as female suicide mortality. Most of the published suicide prevention programmes have in fact been more successful for women than for men [8]–[9], probably due to the different and poorer help-seeking behaviour of men [10].

Several attempts have been made to implement suicide prevention programmes in European countries [11]–[15]. For instance, an educational programme carried out in the Swedish island of Gotland in 1983–1984 to improve general practitioners' (GPs) knowledge of depression was followed by a decrease in suicide mortality in the region [16]–[20]. Most of this decrease could be observed in depression-related suicides among women, perhaps reflecting the specificity of the educational programme and again the poorer help-seeking behaviour of men [8].

A 5-year depression-management educational programme was conducted in Hungary. Education for GPs and their nurses was accompanied by the setting up of a Depression Treatment Clinic and telephone psychiatric consultation service in the intervention region. The GP-based intervention produced a greater decline in suicide rates than in the surrounding county and the country as a whole. An important conclusion of the study was that optimal suicide prevention plans must consider major local risk factors as well [15], [21].

The Nuremberg Alliance Against Depression (NAAD) was an intensive four-level intervention programme carried out in the German city of Nuremberg (about 500,000 inhabitants) in 2001–2002 with the aim to improve the care of depressed people and to prevent suicidality. Concerning the number of suicidal acts (addition of completed and attempted suicides) a significant reduction (24%) was observed during the 2-year intervention period compared to both a baseline year and a control region (the city of Würzburg) [22], an effect that was sustainable during the follow-up year [23]. Suicide rates were not chosen as the primary outcome because power analysis had shown that due to the low base rate and the high annual fluctuations even large reductions would not become statistically significant. Therefore, conclusions were drawn concerning suicidal acts. The assessment of attempted suicides, the use of a control region, the a-priori-defined primary outcome (suicidal acts) and an evaluation plan were the particular strengths of this study.

Since 2003, the NAAD concept and its materials have been adopted and improved by other regions in Germany and in other European countries. This was achieved by the “European Alliance Against Depression” (EAAD, www.eaad.net), launched in 2004 with funding by the European Commission. The basic idea was to refine and complement the intervention concept and material by input from other European countries and to promote the start of regional alliances against depression with a four-level intervention [22], [24]. Meanwhile, more than 100 regions in Europe have started such alliances against depression and the European Commission mentioned EAAD as an example of best practice [25].

The first phase of the EAAD project (2004–2006) set up such programmes in cooperating regions and adapted NAAD concepts and materials to local needs. It was in this context that a 2-year intervention programme was conducted in Szolnok, a town in a region of Hungary where suicide rates are very high. The aim of this study was to evaluate the effectiveness of this two-year, regional, community-based, four-level suicide prevention programme by comparing changes in suicide rates to baseline in the intervention region, a control region, and in the whole country as well.

## Methods

### Ethics Statement

The research was approved by the Semmelweis University Regional and Institutional Committee of Science and Research Ethics, Hungary (ref. TUKEB 23/2005).

### Participants and region

The suicide rate is especially high in the southern and eastern regions of Hungary where the cities Szolnok and Szeged are located. Both cities have a hospital with in- and outpatient psychiatric departments, and were chosen on the criteria of population, infrastructure, and suicide rate, which had been higher than the national average in previous years.

The town of Szolnok, the Hungarian intervention region, had 76,881 inhabitants in 2004, 36,314 men and 40,567 women. The population was essentially stable during the intervention. The unemployment rate was 5.9% in 2004, 6.5% in 2005 and 6.0% in 2006. (The respective national rates were 6.1%, 7.2%, and 7.5%).

As suicide rates in the South-Eastern part of Hungary are historically higher than in other parts of the country [26], Szeged, the biggest city in that area of the country, was chosen as the control region. Since there was no intervention activity in this city, it could be used as a basis for comparison of the change in suicide rates during the intervention period. The city of Szeged had 162,586 inhabitants in 2004 (74,754 men and 87,829 women) . The unemployment rate there was 4.7% in 2004, 5.1% in 2005, and 4.0% in 2006.

### The 4-level intervention concept of the European Alliance Against Depression (EAAD)

**Figure 1:**
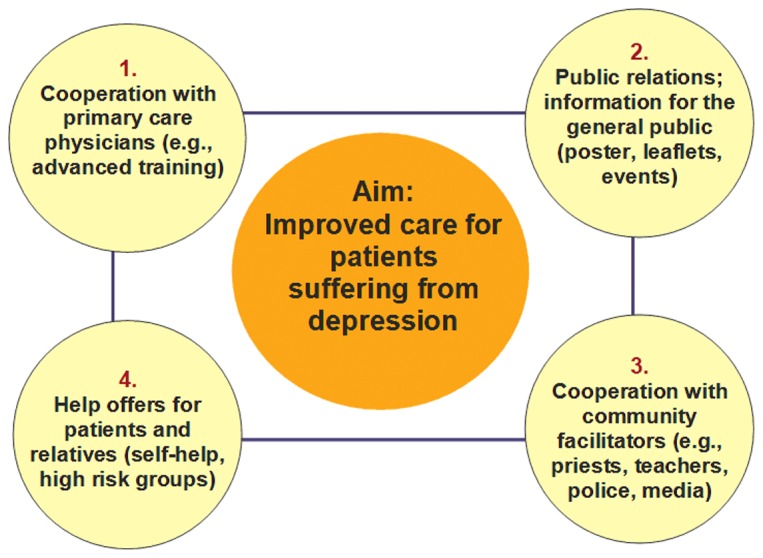
The four-level approach of the European Alliance Against Depression.

EAAD was a project funded by the European Commission, in which 18 European regions worked together to improve the care of people with depression and to prevent suicide across Europe. As shown on Figure 1, the intervention took place on four mutually complementary levels; for details see the works of Hegerl and colleagues [22], [27], [28].

#### Level 1: Co-operation with general practitioners

Interactive workshops using educational packages were developed and offered to GPs. To improve detection of patients with depression, GPs were encouraged to use the shortened Beck Depression Inventory in their practices [29]. To improve treatment utilization, the collaboration between the psychiatric outpatient service and the GPs was strengthened by organizing education programs, panel and roundtable discussions, and setting up an online information centre (www.depressziostop.hu).

#### Level 2: Public relations campaign

The programme started with an opening conference at the town hall for helping professionals and for media workers. 10,000 leaflets and 250 posters were disseminated in Szolnok during the intervention and two publications were released and disseminated on the subject entitled *Together against Depression* [30] and *Depression among children and adolescents* [31]. After the campaign kick-off, press conference, and press release there were 49 subsequent appearances in the media (including TV, radio interviews, articles in local and national newspapers). Twenty-four of these were during the three-week period directly after the press conference but there were also several replays later.

#### Level 3: Community facilitators

In view of the important role of community facilitators, educational workshops were arranged for teachers, district nurses, hotline workers, counsellors, clerics, nurses, policemen, pharmacists and others. These professionals might be influential in depressed and suicidal persons' decisions to access care. Special educational packages were developed for these community facilitators on the following topics: epidemiology, recognition and treatment of suicide risk and depression, depression and anxiety, depression in young and old individuals, the role of different helping professionals in suicide prevention, and suicide risk recognition. During the intervention, 230 community facilitators were trained. There was also close co-operation with the media to promote preventive activities. Media guidelines were handed out recommending how to report on suicides, and how not to report on them in order to avoid imitation suicides.

#### Level 4: High risk groups and self-help

An “emergency card” [32] was produced with an emergency hotline telephone number. The emergency cards were attached to the leaflets with information on facilities such as telephone emergency services, professionals, psychiatrists and relevant local charitable organisations. The leaflets with emergency cards were distributed among the patients of the local psychiatry. A local information data network was built up required for facilitating fast communication on the subject. In addition, educational materials were provided to support the local non-stop telephone emergency services. Head of this latter organization was also involved in the EAAD core group.

### Outcome measures and statistical procedures

Suicide mortality and population data for Hungary and Szolnok were obtained from the Hungarian Central Statistical Office. Reporting of suicides in Hungary is quite accurate because an autopsy is required in every suspected case of suicide. Suicide death rates in the tables and figures of this article were expressed as numbers of deaths per 100,000 men or women.

Suicide rates of the years before the intervention (2002, 2003, 2004) were compared to those during and after the intervention by t-test. The latter period comprised the two intervention years (2005, 2006) and the follow-up year of 2007. The first follow-up year was included because effects of the intervention can be expected not to stop with the official end of the intervention and effects sustainable in the first follow-up year have been reported in EAAD. Number of visits to the psychiatry department in Szolnok and the number of calls to the local emergency hotline in the region were also investigated as secondary outcome measures.

Generalized mixed linear models (GLIMMIX) were computed in order to detect effects of the intervention on the frequency of suicides. A negative binomial distribution for modelling count variables and a log-link function were chosen for this analysis. “Intervention region” (yes versus no) was selected as a random variable; the fixed main variable was “time” (before versus after the onset of the intervention) in the model. The repeatedly measured dependent variable was the annual rate of suicides per 100,000 persons (2002–2007), with 2002-2004 representing the pre-intervention period and 2005–2007 being the period after the onset of the intervention. In this context, a significant interaction between intervention and time signifies that the changes of the annual suicide rates were different between the intervention and control regions. These analyses were conducted for the total sample. Analogously, the effects of the intervention were compared with the Hungarian suicide trend.

## Results

### Suicide rates and trends

Suicide rates before and after the start of the intervention are shown in Table 1 and 2. During the intervention period, the annual suicide rate in Szolnok decreased from 30.1 per 100,000 inhabitants in 2004 to 13.2 in 2005, 14.6 in 2006 and remained as low as 12.0 in 2007, the first follow-up year after the end of the intervention. In the control region, suicide rates somewhat increased during the intervention period. Although national suicide rates continuously decreased during this period, the overall decline was substantially smaller than in the intervention region. In addition, the decrease in suicide rates in the intervention region was pronounced for both males and females.

**Table 1:** Occurrence and rate (N / 100,000 persons) of suicide in Szolnok, Szeged, and entire Hungary.

	Szolnok (intervention region)						Szeged (control region)						Hungary (whole country)					
	Men		Women		Total		Men		Women		Total		Men		Women		Total	
Year	N	Rate	N	Rate	N	Rate	N	Rate	N	Rate	N	Rate	N	Rate	N	Rate	N	Rate
2002	19	52.65	6	14.62	25	32.42	37	48.97	14	15.88	51	31.15	2 195	45.47	648	12,16	2 843	27,99
2003	14	38.97	7	17.13	21	27.35	31	41.32	9	10.25	40	24.56	2 161	44.92	640	12,03	2 801	27,65
2004	16	44.83	7	17.16	23	30.08	25	33.44	12	13.66	37	22.76	2 087	43.49	655	12,34	2 742	27,13
**2005**	**7**	**19.76**	**3**	**7.38**	**10**	**13.15**	**28**	**37.47**	**12**	**13.61**	**40**	**24.56**	**2 028**	**42.35**	**593**	**11,19**	**2 621**	**25,98**
**2006**	**6**	**17.03**	**5**	**12.38**	**11**	**14.55**	**32**	**42.68**	**13**	**14.73**	**45**	**27.56**	**1 861**	**38.92**	**600**	**11,34**	**2 461**	**24,44**
2007	6	17.12	3	7.46	9	11.96	38	50.24	8	8.96	46	27.90	1 879	39.36	571	10,81	2 450	24,36

Values containing data from the time interval of the intervention are bold.

**Table 2:** Comparison of suicide rates from the three baseline and intervention years.

		Szolnok (intervention region)			Szeged (control region)			Hungary (whole country)		
		Men	Women	Total	Men	Women	Total	Men	Women	Total
2002–2004	M	45.49	16.30	29.95	41.25	13.26	26.16	44.62	12.18	27.59
	SD	6.86	1.46	2.54	7.77	2.84	4.42	1.02	0.15	0.43
2005–2007	M	17.97	9.08	13.22	43.46	12.43	26.67	40.21	11.12	24.93
	SD	1.55	2.86	1.29	6.42	3.06	1.84	1.87	0.27	0.92
Mean change		−60.5%	−44.3%	−55.9%	5.4%	−6.3%	2.0%	−9.9%	−8.7%	−9.6%
t-values		6.9451*	8,5973*	11,4123**	−0,4948	0,5067	−0,2019	7,5073*	11,9771**	10,6742**
Cohen's d		5.53	3.19	8.30	0.31	0.28	0.15	2.94	4.78	3.72

*p<.05; **p<.01.

Unemployment is a crucial factor in the background of suicide. Therefore, we also investigated whether this variable could explain the observed decrease of suicide rates. The unemployment rate in Szolnok was 5.9% in 2004, somewhat lower than the national rate (6.1%), and increased to 6.5% in 2005 and 6.0% in 2006. The Spearman's rank correlation between unemployment rate and suicide rate was 0.15 (p = 0.65) in the intervention region between 1998 and 2008.

The GLIMMIX results revealed significant interactions of the factors “region” and “time” (2002–2004 versus 2005–2007): In the intervention region, the decline of annual suicide rates was significantly more pronounced than in Hungary (F_(1,8)_ = 6.57, p = .017, one-sided test) and in the control region (F _(1,7)_ = 20.85, p = .0015, one-sided test).

**Figure 2:**
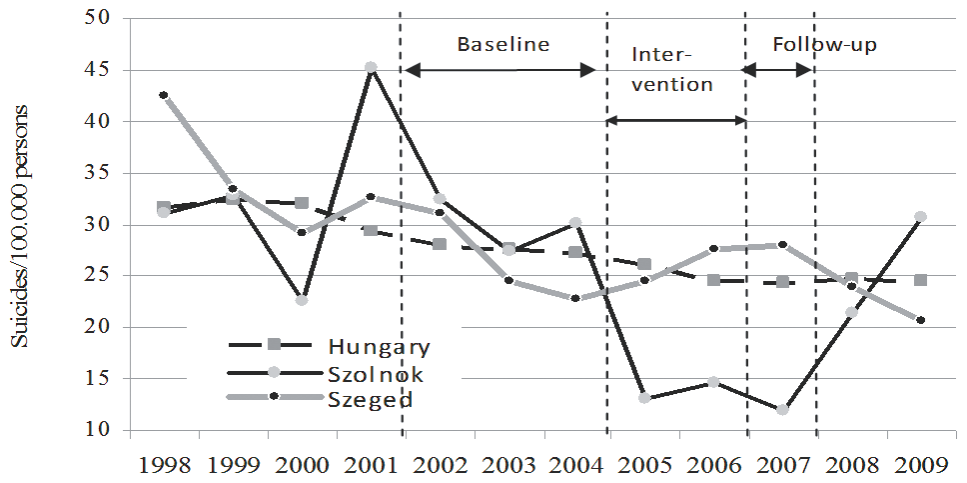
Suicide rates in Szolnok, Szeged, and Hungary (1998–2009).

In figure 2, the suicide rates are shown not only for the periods considered for the statistical analysis (three baseline years, three years after start of the 2-year intervention) but also for a more extended time period. The data show that two and three years after the end of the intervention (2008 and 2009), suicide rates returned back to the higher pre-intervention level in Szolnok.

### Secondary outcome measures

**Figure 3:**
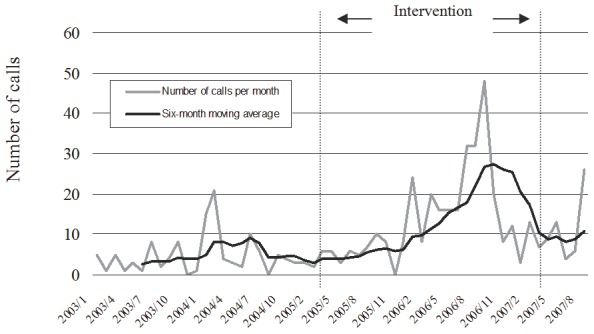
Number of telephone calls with suicide problems to the hotline service in Szolnok between 2003 and 2007.

The number of calls to the emergency hotline service, especially those involving suicide problems, increased significantly between the periods 2003–2004 and 2005–2007. Figure 3 shows the number of calls with suicide problems to the hotline service in Szolnok. The number of outpatient visits to the psychiatric clinic in Szolnok also increased from 2,520 at baseline in 2004 to 3,147 (+24.9%) in the first intervention year and 4,431 (+75.8%) in the second [33].

## Discussion

The most important outcome of the EAAD programme [32] in Hungary was that after the start of the intense four-level intervention in Szolnok, a significantly stronger decrease in suicide rates was observed in this region (56%) compared to overall national trends (10%) or the slight *increase* (2%) observed in the control region of Szeged. The decrease in the intervention region was similar for both males and females. This latter finding may be exceptionally relevant, since other programmes have shown substantially lower benefits for men than for women [8], [9].

One possible explanation of the greater benefit for men in the intervention region might be the anonymity of the emergency hotline service, which seems to be more appropriate for men. The programme incorporated intensive promotion of the anonymous emergency hotline services and as a result, the number of emergency calls, especially those concerning suicide-related problems, increased substantially. Another possible factor could be that while training of GPs alone will not be as helpful for male patients as for women who tend to seek professional help much more readily than men. The public relation campaign of the four-level intervention might have promoted male help seeking particularly.

However, the large increase in emergency calls indicates that the four-level intervention had an overall facilitating effect on help seeking behaviour of people with suicidal tendencies. This is further supported by the substantial increase in patient visits to the psychiatric outpatient department in the local hospital. Motivation for seeking psychiatric help is usually higher among women, but effective hotline advice as a first step intervention could motivate men to utilise non-anonymous services as well, mostly psychiatric care.

The present study has several limitations that need to be taken into account before drawing conclusions. The magnitudes of the effects are numerically correct, but have to be interpreted with caution in view of the small sample sizes. Also, such community-based interventions, although controlled for general trends in suicide rates in the whole population and in a control city, do not provide proof for efficacy with the same evidence level as a randomized controlled study. Besides random fluctuations, there are too many factors which are hard to control. However, whereas the effectiveness of the four-level intervention concept has already been demonstrated concerning suicidal acts (attempted + completed suicides) within the Nuremberg Alliance against Depression project, this study seems to provide evidence for the effectiveness of this approach concerning suicides.

In our study, it is not possible to draw conclusions as to which elements of the four-level intervention might have been the most relevant. Such inferences would be especially difficult to make because of the strong synergistic effects developed by being simultaneously active on four levels. For example, the public relations campaign might have motivated depressed people to seek help from their GP and the GP might have been motivated by that to increase his competence in treating depression.

For a more profound evaluation, it will be necessary to identify some other mediating variables. Future research should assess health behaviour (notably alcohol and psychoactive agent use) and gather additional data about health care utilization (e.g. prescription rate of antidepressants). Since the suicide rate is traditionally high in Hungary and seems to be quite independent of major economic and political changes, the evaluation of cultural background (e.g. public attitudes to suicide) would also be important.

Since suicide rates may have random fluctuations (especially in small populations), a longer observation period may increase the strength of our study. Nonetheless, our findings may prompt further prospective and more targeted studies to answer these questions.

## Conclusions

Results of the present study seem to provide further support for the effectiveness of the four-level community-based intervention concept concerning prevention of suicidal behaviour in both sexes. Sustainability of improvements should be a key point in future research and its implementation.
